# Short-term response of the soil bacterial community to differing wildfire severity in *Pinus tabulaeformis* stands

**DOI:** 10.1038/s41598-019-38541-7

**Published:** 2019-02-04

**Authors:** Weike Li, Shukui Niu, Xiaodong Liu, Jianming Wang

**Affiliations:** Beijing Key Laboratory of Forest Resources and Ecosystem Process, College of Forestry, Beijing Forestry University, Beijing, 100083 China

## Abstract

In recent years, the investigation of fire disturbance of microbial communities has gained growing attention. However, how the bacterial community varies in response to different severities of fire at different soil depths is largely unknown. We utilized Illumina MiSeq sequencing to illustrate the changing patterns of the soil bacterial community following low-, moderate- and high-severity wildfire in the topsoil (0–10 cm) and subsoil (10–20 cm), 6 months after the fire. Acidobacteria, Proteobacteria, Actinobacteria, Verrucomicrobia and Chloroflexi were the dominant phyla among all samples. Bacterial alpha diversity (i.e. Shannon and Simpson indices) in the topsoil was significantly higher than that in the subsoil after a high-severity wildfire. Non-metric multidimensional scaling (NMDS) analysis and permutational multivariate analysis of variance (PERMANOVA) revealed significant differences in the bacterial community structure between the two soil layers. Soil pH, ammonium nitrogen (NH_4_^+^-N) and total nitrogen were the main factors in shaping the bacterial community structure, of which soil pH was the most robust in both soil layers. Our study reveals that wildfire results in short-term changes in soil bacterial community. However, a long-term monitoring of microbial variation after burning is also essential.

## Introduction

Fire is a common environmental perturbation in forest ecosystems^1^. There are around 12,670 fires per year on average in China, and the burned area was about 604,126 ha during each of the last six decades^2^. Fire produces a broad spectrum of effects, which depend on vegetation type, topography, fuel load and combustion, soil, climate and duration^3^. The effects of fire can lead to major shifts in a variety of environmental parameters that are likely to have large direct and indirect effects on soil microbial community composition and diversity^4^. Therefore, understanding the potential ecological effects of fire is critical for the succession and restoration of burned stands. Prior studies have revealed the alterations in soil physicochemical properties caused by wildfires, including consumed organic matter^5^, destabilized soil structure^6^, decreased water infiltration and increased soil erosion with the formation of hydrophobic layers following a fire^7,8^, as well as an increase in soil pH^9,10^. Additionally, wildfire often causes an immediate loss of soil organic nitrogen (N) through volatilization or mineralization^11^. There is usually a pulse of ammonium nitrogen (NH_4_^+^-N) and nitrate nitrogen (NO_3_^−^-N) following a fire^12^. Ammonium is the predominant direct product of the combustion, and is subsequently transformed to nitrate as a result of nitrification^9,13^. Both ammonium and nitrate are available biologically to soil microorganisms, but if not taken up immediately, large fluxes from ecosystems may occur.

Soil microbes play an important role in ecosystem recovery. They can be affected by fire, either as a direct result of heating or as an indirect effect of changes in soil physicochemical properties^14^. Several studies have shown the association between wildfire and microbial communities^15,16^. Neary *et al*.^17^ reported that heating effects on microbes are most significant in the topsoil where microorganisms are most abundant. According to Hamman *et al*.^18^, although microbial biomass did not change after burning, microbial community structure was different in areas with differing fire severity. Microbes differ in their sensitivity to fire-induced heat^19^. It has been reported that bacteria are more resistant to heat disruption than fungi and generally recover more rapidly after a fire^20^. This may be explained by high pH favouring bacterial growth^21,22^ and increased concentrations of dissolved organic matter resulting from the wildfire^23^. Although many field studies have investigated the responses of soil microbes to forest fire in boreal and tropical regions^3,24–26^, little is still known about the effects of wildfire on the soil bacterial community in temperate coniferous forests.

Wildfires are often extremely heterogeneous, depending on fuel distribution and wind direction and strength, which results in large patches of unburned vegetation and areas experiencing differing fire severity within the boundaries of the fire. Additionally, temperature profiles in the soil vary with fire severity. Mineral soil temperatures cannot usually exceed 50 °C at 5 cm depth after a low-severity fire^27^. However, temperatures of 100 °C were observed as far as 22 cm below the ground surface where severe soil burning occurred^17^. The belowground microbial community structure of the unburned patches is potentially very different from that of burned areas^28^. In this study, we examined the effects of different fire severities (high, moderate, low and unburned) on soil bacteria at two soil depths (0–10 cm topsoil and 10–20 cm subsoil) in a *Pinus tabulaeformis* forest in northern China.

Our major hypotheses were that:

1. The bacterial community composition will vary differently in the topsoil and subsoil after burning at different severities. It is expected that the bacterial community in the topsoil will showed a higher diversity.
2. The main driving factors for the topsoil and subsoil bacterial community composition after a wildfire are different.

## Results

### Soil physicochemical characteristics

As shown in Table S1, soil physicochemical properties were altered by the wildfire. The lowest values for soil organic matter (OM), total nitrogen (TN), Ammonium nitrogen (NH_4_^+^-N) and nitrate nitrogen (NO_3_^−^-N) were found in the topsoil and subsoil of the areas affected by high-severity wildfire. Contrary to the above soil properties, soil pH was increased after burning, with the highest values detected in the high-severity wildfire areas (*P* < 0.05). Interestingly, soil moisture (SM) was decreased after moderate- or low-severity wildfire; however, it was increased significantly in the high-severity wildfire areas.

### Sequencing results

**Figure 1:**
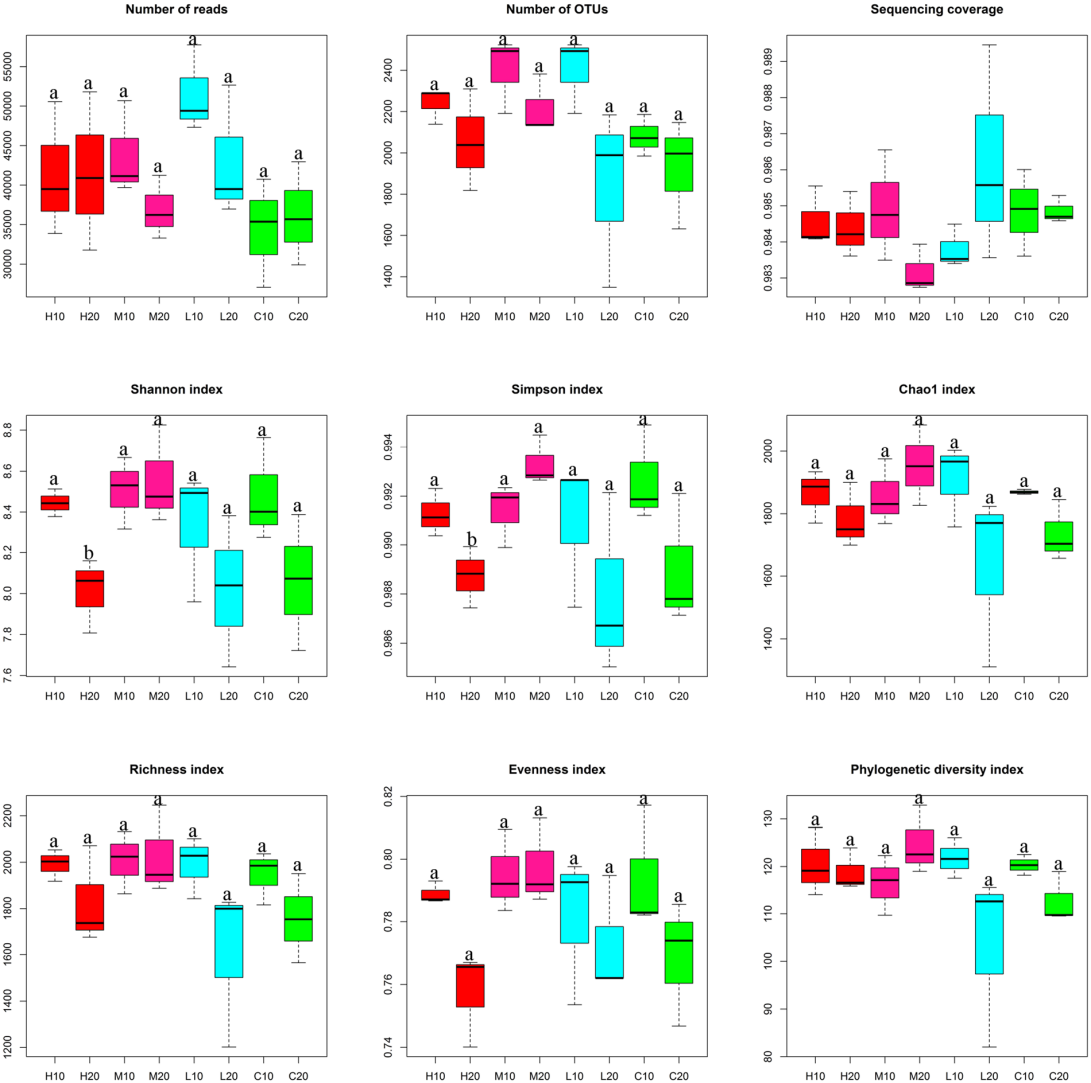
Sequencing results and bacterial alpha-diversity in the soils of areas affected by differing fire severity. Different lowercase letters indicate a significant difference between samples in the topsoil and subsoil following a fire of the same severity (*P* < 0.05). H, high severity; M, moderate severity; L, low severity; C, unburned. 10, topsoil (0–10 cm); 20, subsoil (10–20 cm).

A total of 986,036 reads and 4,665 operational taxonomic units (OTUs) after quality filtering were generated from 24 soil samples through Illumina MiSeq sequencing analysis. Each library contained 27,061 to 57,755 reads, with the number of OTUs ranging from 1,349 to 2,523 (Fig. 1). Independent-sample *t*-tests indicated that there was no significant difference in the number of bacterial reads or OTUs between the topsoil and subsoil in areas affected by fire of differing severity (Fig. 1). The sequencing depth index (coverage) of all samples was above 0.97, indicating that the sequencing could meet the requirements of the analysis (Fig. 1).

**Figure 2:**
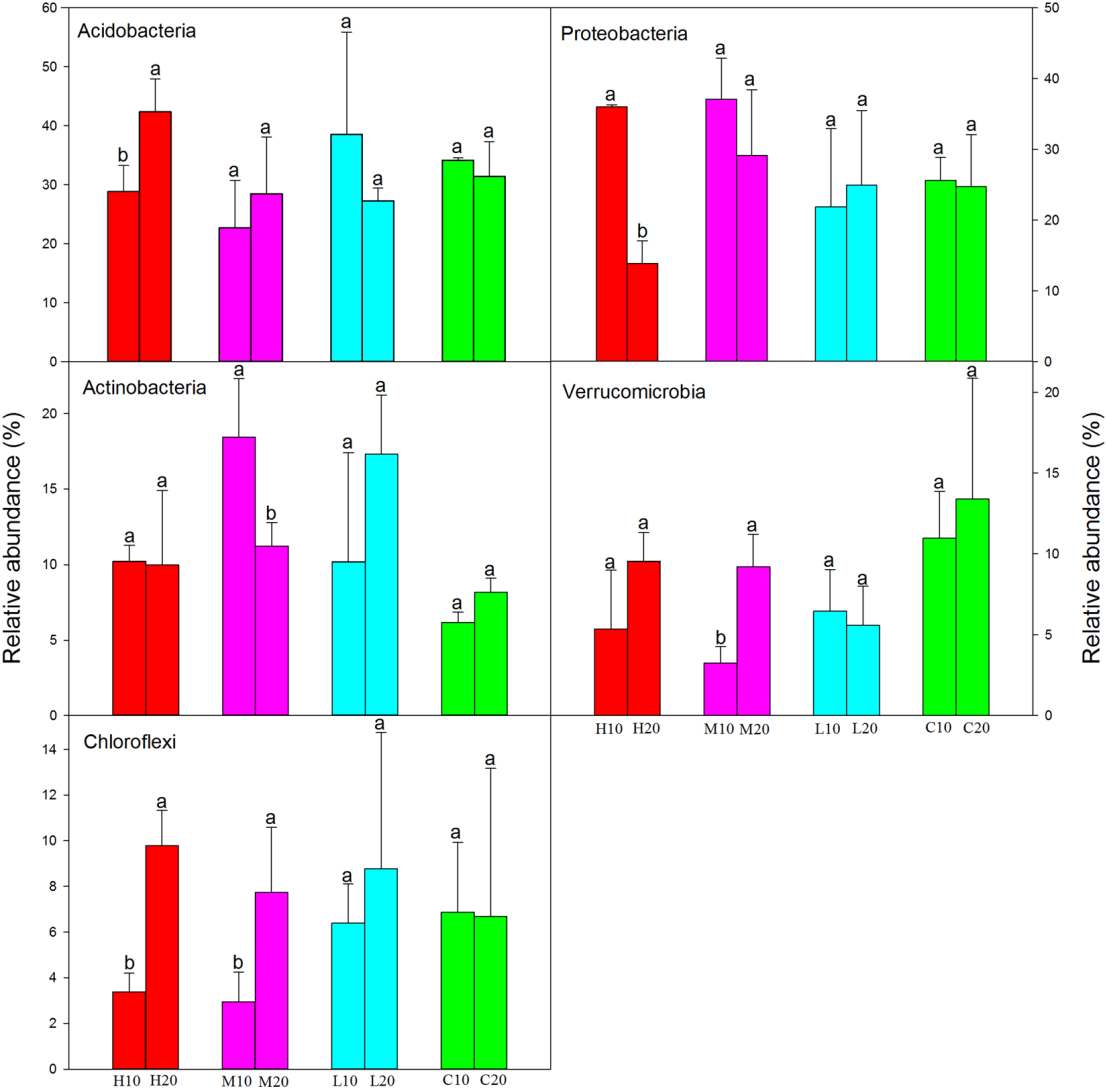
Relative abundance of the dominant bacterial phyla in areas affected by differing fire severity. Different lowercase letters indicate a significant difference between samples from the topsoil and subsoil following a fire of the same severity (*P* < 0.05).

**Figure 3:**
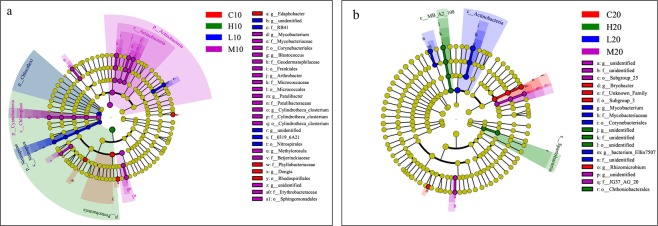
The cladogram showed the most differentially bacterial taxa (from the phylum level as the innermost cycle to genus level as the outermost circle) within the topsoil (**a**) and subsoil (**b**). The yellow nodes indicate no statistically significant differences of bacterial taxa among different groups. The red, green, blue and purple nodes indicate statistically significant differences of bacterial taxa in the corresponding group from other groups.

The bacterial sequencing reads belonged to 40 different phyla, 80 classes, 139 orders, 221 families and 293 genera. Among these phyla, *Acidobacteria* (31.77%), *Proteobacteria* (26.59%), *Actinobacteria* (11.80%), *Verrucomicrobia* (7.64%) and *Chloroflexi* (6.60%) were dominant (relative abundance >5%) and common to the 24 libraries (representing the 24 soil samples), jointly accounting for 84.40% of the total reads (Fig. 2). We did a linear discriminant analysis effect size to identify the classified bacterial taxa differences among different fire severity areas within the topsoil and subsoil (Figs 3 and S2). The result indicated that 36 and 21 bacterial clades presented significantly different with a LDA threshold of 2.0 in the topsoil and subsoil respectively (Figs 3 and S2). These bacterial taxa could be considered as the biomarkers in the corresponding area.

Fire changed the proportion of some dominant bacterial phyla in the topsoil and subsoil. The topsoil showed significantly higher relative abundances of *Proteobacteria* and *Actinobacteria*, but significantly lower relative abundances of *Acidobacteria*, *Verrucomicrobia* and *Chloroflexi* after high- or moderate-severity wildfire (Fig. 2). However, no significant differences in abundance of dominant bacterial phyla between the two soil layers were found in the low-severity wildfire or unburned areas (Fig. 2). SIMPER analysis was used to identify which bacterial phylum made the greatest contribution to the community dissimilarity between the topsoil and subsoil. The result showed that although *Proteobacteria* was the second most abundant bacterial phylum, it made a greater contribution than any other bacterial phyla to the differences in community composition between the topsoil and subsoil (Table 1). This indicated that there was no correlation between the abundance of bacterial phyla and their contribution to community dissimilarity.

**Table 1:** Contribution of dominant soil bacterial phyla to differences in community composition between the topsoil and subsoil.

Dominant bacterial phyla	The contribution of bacterial phyla (%)	
	Average	Standard deviation
Proteobacteria	5.72	0.04
Acidobacteria	5.04	0.04
Actinobacteria	2.95	0.02
Verrucomicrobia	2.39	0.02
Chloroflexi	2.11	0.02

### Bacterial community diversity and structure

A randomly selected subset of 27,061 bacterial sequences per sample was used for alpha-diversity analysis. We found that the Shannon and Simpson indices of bacterial diversity in the topsoil were significantly higher than those in the subsoil after high-severity wildfire (*P* < 0.05) (Fig. 1). However, there were no significant differences in the Chao1, Richness, Evenness or Phylogenetic diversity indices between the two soil layers in areas affected by wildfire of different severities (*P* > 0.05) (Fig. 1).

**Figure 4:**
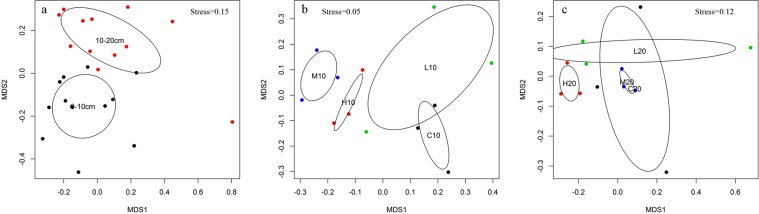
NMDS showing differences in bacterial community structure according to Bray–Curtis distance. (**a**) Total samples; (**b**) samples from topsoil; (**c**) samples from subsoil.

Non-metric multidimensional scaling (NMDS) analysis showed that the bacterial community structures of topsoil and subsoil could be clearly divided (Fig. 4a). The permutational multivariate analysis of variance (PERMANOVA) also confirmed that the bacterial community in the topsoil was significantly different form that in the subsoil (R^2^ = 0.07, *P* = 0.04) (Table 2). Further analysis was conducted among the topsoil and subsoil samples separately. Although we could see that several groups could be well separated from the others, for example, groups H10 and H20 were clearly distinct from the other groups in the topsoil and subsoil, respectively, PERMANOVA analysis demonstrated that these differences were not significant (*P* > 0.05) (Fig. 4b,c, Table 2).

**Table 2:** Differences in bacterial communities examined by permutational multivariate analysis of variance (PERMANOVA).

PERMANOVA					
	*R* ^2^	P value		*R* ^2^	P value
Topsoil	0.07	0.04	H10 *vs*. M10	0.35	0.1
			H10 *vs*. L10	0.27	0.2
			H10 *vs*. C10	0.37	0.1
			M10 *vs*. L10	0.32	0.1
			M10 *vs*. C10	0.47	0.1
			L10 *vs*. C10	0.21	0.3
Subsoil			H20 *vs*. M20	0.46	0.1
			H20 *vs*. L20	0.30	0.1
			H20 *vs*. C20	0.35	0.1
			M20 *vs*. L20	0.24	0.4
			M20 *vs*. C20	0.24	0.2
			L20 *vs*. C20	0.18	0.6

H, high severity; M, moderate severity; L, low severity; C, unburned. 10, topsoil (0–10 cm); 20, subsoil (10–20 cm).

### Correlation between bacterial community structure and soil variables

**Figure 5:**
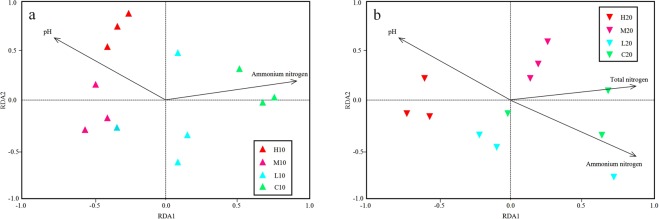
RDA of bacterial community composition and soil variables. (**a**) Samples from topsoil; (**b**) samples from subsoil.

Redundancy analysis (RDA) was performed to determine the relationship between soil properties and the bacterial community. In both the topsoil and subsoil, we found that pH and NH_4_^+^-N strongly influenced the bacterial community distribution (Fig. 5a,b). TN also had a significant effect on the bacterial community structure in the subsoil (Fig. 5b). A variance partitioning analysis (VPA) was conducted to quantify the relative contributions of the soil properties to the bacterial community structure. The soil variables selected explained a total of 56.93% and 42.70% of the community variation in the topsoil and subsoil, respectively. Among these variables, soil pH explained the most variation in both soil layers (12.64% in topsoil; 17.48% in subsoil) (Table 3). In addition, soil pH also showed a significant correlation with the relative abundance of some dominant phyla, such as *Verrucomicrobia* and *Chloroflexi* in the topsoil and *Acidobacteria* and *Proteobacteria* in the subsoil (Table S2). These results strongly indicated that the bacterial communities from the different soil layers were affected by pH, NH_4_^+^-N and TN, and that soil pH occupied a central role in shaping the structure of the bacterial community after a wildfire.

**Table 3:** Variance partitioning analysis of the contribution (percentage) of soil properties to bacterial community structure in the topsoil and subsoil after a wildfire.

Indices	Explained (%)	
	Bacteria in topsoil	Bacteria in subsoil
OM	9.88	4.13
TN	9.00	5.71
NH_4_^+^-N	10.33	5.80
NO_3_^−^-N	7.69	3.97
SM	7.39	5.61
pH	12.64	17.48
Total	56.93	42.70

## Discussion

Fire plays an important role in the terrestrial ecosystem, and the effects of fire on soil physicochemical characteristics have been studied extensively^29^. Consistent with previous research^16,29–32^, we found similar results in this study (Table S1). Wildfire altered the content of C and N in soil, which decreased with increasing fire severity (Table S1). Soil pH is generally considered to rise after a fire^9^, which was also found in our study. This may be due to the effect of fire on the oxidation of surface litter, which causes a release of soluble base and cations in the soil^18^. Interestingly, the water content of the topsoil was found to be highest in areas affected by high-severity fire. This phenomenon was probably attributed to the destruction of the hydrophobic layer^7^, as well as the complete disappearance of the soil litter layer and a decline in the ability to intercept rainwater after the death of the aboveground vegetation^18^, resulting in a significant increase in the soil moisture content after a high-severity burning.

Not entirely consistent with our first hypothesis. We found no significant differences in the number of sequencing reads or OTUs between the two soil layers in areas affected by fire of differing severity (Fig. 1). However, the α-diversity (Shannon and Simpson indices) of topsoil bacteria was significantly higher than that of subsoil bacteria in the area affected by high-severity wildfire (Fig. 1). This was inconsistent with the results of Scott *et al*.^4^, who observed a significant reduction in the α-diversity of soil bacteria 4 and 16 weeks after a severe wildfire. However, Shen *et al*.^33^ reported a significant increase in bacterial α-diversity in the topsoil after a treatment of burning every 2 years. The reason for these differences might be the different sampling times and forest land environments^3,18^. We speculated that soil bacterial diversity could recover in as little as a growing season with fast division and colonization^34^.

*Acidobacteria*, *Proteobacteria*, *Actinobacteria*, *Verrucomicrobia* and *Chloroflexi* were found to be dominant bacterial phyla in this study (Fig. 2), similar to the observation from soils collected in a Chinese boreal forest^3^. The relative abundance of some bacterial phyla was changed significantly by the wildfire (Fig. 2). For example, consistent with results of most previous studies, *Acidobacteria* showed a significant decline in the topsoil after a high-severity fire^35,36^. However, a higher relative abundance of *Acidobacteria* was found in the subsoil, which might be due to the oligotrophic environment^37^. We also found a significantly lower abundance of *Proteobacteria* in the subsoil than that in the topsoil after a high-severity fire. A previous study reported a high abundance of *Proteobacteria* in the rhizosphere soil and a close symbiotic relationship between the phylum *Proteobacteria* and plant roots^38^. High severity fires can cause death of aboveground vegetation as well as destruction of plant roots. Therefore, this might be an important reason for the difference in the abundance of *Proteobacteria* between the topsoil and subsoil, but additional analyses based on rhizosphere and non-rhizosphere soil bacteria would be necessary to test this hypothesis.

NMDS and PERMANOVA analysis further revealed that soil bacterial communities in the topsoil were significantly different from those in the subsoil (Fig. 4, Table 2). In order to determine the reasons for these differences, but also to answer our second hypothesis, we performed RDA and VPA analyses. The results indicated that soil pH, NH4^+^-N and TN were the main driving factors of bacterial community change in our study (Fig. 5, Table 3). Accumulating literature has proven that soil pH is a universal predictor of differences in microbial community distribution, including bacteria^39^, fungi^23^ and several specific taxa^40^. Meanwhile, soil pH has also been demonstrated to be an important factor affecting the composition of bacterial communities across a variety of spatial scales, from continents to small and sub-metre scales^3,36,41,42^. However, most of the extant research on the responses of the microbial community to wildfire (or prescribed fire) has been performed using surface soil, which is generally considered the most sensitive to fire^43^. In this study, we found that pH was a robust factor for determining bacterial community composition, not only in the topsoil, but also in the subsoil (Fig. 5, Table 3). Interestingly, for some dominant phyla, the trend of variation with pH in different soil layers was not consistent. For example, in the topsoil, the relative abundance of *Acidobacteria* showed a negative correlation with elevation of soil pH (Table S2), which was consistent with results of other studies^33,42^, whereas a positive correlation between the percentage of *Acidobacteria* and soil pH was found in the subsoil (Table S2). This might be due to the different response of *Acidobacteria* subgroups to soil pH^44^.

Besides pH, bacterial community structure was also influenced significantly by NH_4_^+^-N and TN (Fig. 5). Saetre and Bååth^45^ reported that the content and availability of carbon and nitrogen in soil can directly affect the structure and function of the soil microbial community. Zhao *et al*.^46^ observed that an increase in TN could promote the growth and reproduction of *Chloroflexi*, which is consistent with the significant positive correlation between TN and *Chloroflexi* abundance found in this study (Table S2). NH_4_^+^-N is an important nitrogen source for microbial growth, and a change in its content will inevitably affect the growth and reproduction of some NH_4_^+^-N-sensitive microbial groups^47^. Zhou *et al*.^48^ found that an increase in the relative abundance of *Acidimicrobiia*, *Alphaproteobacteria* and *Gammaproteobacteria* was related to an improvement in NH_4_^+^-N. However, we found that only *Verrucomicrobia* were strongly affected by NH_4_^+^-N in the topsoil (Table S2), implying that although NH_4_^+^-N was significantly correlated with bacterial community structure, it was not a universal predictor of bacterial taxa distribution in our study.

VPA analysis showed that nearly half of the variation in bacterial community composition between topsoil and subsoil was not explained in this study. Besides the influence of soil properties, microbial community structure is also affected by vegetation composition^49^, climate change^50^ and other factors^51,52^. The influence of these factors on soil microbial community and the relationship between these factors and microorganisms needs further study. However, the recovery of soil microbial communities after fire is a continuous and dynamic process and long-term monitoring of microbial variation is essential.

## Materials and Methods

### Study area

The study area was located in Pingquan County, Hebei province, northern China (118°22′~118°37′E, 41°01′~41°21′N) (Fig. S1). The climate in this region is semi-humid and semi-humid continental monsoon, with an annual mean temperature of 7.3 °C and annual precipitation of 540 mm. The dominant tree species in this area is secondary *Pinus tabulaeformis* (a forest which has re-grown after being deforested), and the soil types are mainly brown soil and cinnamon soil^53^. In April of 2015, a 56.33 ha wildfire occurred in this region. The study area was classified into different sites according to fire severity (high-severity, moderate-severity, low-severity and nearby unburned sites). Severity was determined by percentage tree mortality and some visual indicator such as char height of trunks, survival of undergrowth shrubs or soil colour^54^. In brief, sites affected by high-severity wildfire had more than 80% tree mortality while those affected by low-severity wildfire had less than 10% tree mortality. The char height of trunks was more than 5 m at high-severity sites but less than 2 m in low-severity areas. Values of these indicators for moderate fire severity were between those measured at the high- and low-grade fire sites (Table S3). There was no significant difference among all sites in terms of slope (20~23°), aspect (shady) or elevation (1119~1143 m).

### Soil sampling and analysis

Soils were sampled in October 2015. Three separate plots in each of the burned and unburned areas, measuring 20 × 20 m, were established randomly. In each plot, the litter, charred debris and ash layer were all removed, and then composite soil samples were collected from depths of 0–10 cm and 10–20 cm using a soil corer (5 cm in diameter). Each composite sample was bulked from five random soil cores of the same plot to generate 24 composite samples (12 topsoil; 12 subsoil). All samples were sieved through 2 mm mesh and taken to the laboratory in an ice box for further analysis. Samples were divided into two parts; one part was stored at 4 °C for biogeochemical analysis, and the other was stored at −20 °C for DNA analysis.

Soil moisture was determined by oven-drying the samples at 105 °C until constant weight. pH was determined using a pH meter with a 1:2.5 ratio of fresh soil to deionized water at 20 °C. Soil organic matter content was measured by dichromate oxidation^29^, and TN was measured by the Kjeldahl method^55^. The NH_4_^+^-N and NO_3_^−^-N contents were also measured^56,57^.

### DNA extraction and PCR amplification

Genomic DNA was extracted from 0.5 g fresh soil using an E.Z.N.A.® soil DNA kit (Omega Bio-tek, Norcross, GA, USA), following the manufacturer’s instructions. The quality of DNA extracted was checked by 1% agarose gel electrophoresis and spectrophotometry (ratio of optical density at 260 nm/280 nm). All DNA samples were stored at −20 °C for further analysis.

To assess the bacterial composition of samples, the V3–V4 hypervariable region of the bacterial 16 S rRNA gene was amplified by PCR (95 °C for 5 min, 33 cycles at 95 °C for 30 s, 56 °C for 30 s and 72 °C for 40 s, with a final extension of 72 °C for 10 min), using the universal primers forward 338 F (5′-ACTCCTACGGGAGGCAGCAG-3′) and reverse 806 R (5′-GGACTACHVGGGTWTCTAAT-3′)^51^. These primers contained an 8-nucleotide barcode sequence unique to each sample. PCR reactions were performed in triplicate in a 50 μL mixture containing 5 μL of 10× Pyrobest Buffer, 4 μL of 2.5 mM dNTPs, 2 μL of each primer (10 μM), 0.3 μL of Pyrobest DNA Polymerase (2.5 U/μL; TaKaRa, Code: DR005A) and 30 ng of template DNA. The PCR products were then sequenced. The raw reads were deposited into the NCBI Sequence Read Archive database (Accession Number: SRP158101).

### Illumina MiSeq sequencing

Amplicons were extracted from 2% agarose gels, purified using an AxyPrep DNA Gel Extraction kit (Axygen Biosciences, Union City, CA, USA) according to the manufacturer’s instructions, and quantified using QuantiFluor^TM^-ST (Promega, Madison City, WI, USA). Purified amplicons were pooled in equimolar amounts and paired-end sequenced (2 × 300 bp) on an Illumina MiSeq platform (Beijing Allwegene Technology Co., Ltd, China) according to standard protocols.

### Processing of sequencing data

The extraction of high-quality sequences was firstly performed with the QIIME package (Quantitative Insights Into Microbial Ecology) (version 1.2.1) (http://qiime.org/). Raw sequences were selected on the basis of sequence length, quality, primer and tag. Low-quality sequences were removed as follows: (1) 300 bp reads were truncated at any site receiving an average quality score of <20 over a 10 bp sliding window, and truncated reads shorter than 50 bp were discarded; (2) only a 2-nucleotide mismatch in primers was allowed, and reads containing ambiguous characters were removed; (3) only sequences with overlap longer than 10 bp were assembled according to their overlap sequence. Reads which could not be assembled were discarded. The unique sequence set was classified into OTUs under the threshold of 97% identity using UCLUST^3^. Chimeric sequences were identified and removed using Usearch (version 8.0.1623) (http://www.drive5.com/usearch/). The taxonomy of each 16 S rRNA gene sequence was analysed by UCLUST against the Silva119 16 S rRNA gene database using a confidence threshold of 90%. OTUs with less than five reads were removed to reduce the risk of artificially inflating richness due to sequencing errors^58^.

### Data analysis

One-way analysis of variance (ANOVA) with Tukey’s test was performed to detect the effects of wildfire of varying severity on soil properties at different soil depths (0–10 and 10–20 cm). The differences of bacterial community abundance and diversity between the topsoil and subsoil were compared by independent-sample *t*-test. Pearson correlation analysis was used to determine relationships between the dominant phyla and soil variables. Differences were considered significant with a *P* value of 0.05 as the threshold. All these tests were carried out using SPSS 19.0 for Windows.

To correct for sampling efficiency, we used a randomly selected subset of 27,061 bacterial sequences (the minimum sample size among 24 samples) per sample for downstream analysis. Several indices (Shannon, Simpson, Chao 1, Richness, Evenness and Phylogenetic diversity index) were calculated using the OTUs with 97% identity to determine bacterial α-diversity. SIMPER analysis was conducted to identify the contribution of differences in the dominant bacterial phyla to the overall community differences. NMDS analysis based on Bray–Curtis dissimilarity and PERMANOVA were used to compare the bacterial community compositions within different fire-damaged areas. RDA was carried out to assess the relationships between soil properties and bacterial community data (relative abundance of OTUs), and only environmental factors with variance inflation factor (VIF) values of <20 were selected to be visualized^3^. VPA was further performed to quantify the effects of soil properties on bacterial community structure. The above analyses were carried out by the Vegan package (version 2.4.2) in R (version 3.3.1) (https://www.r-project.org/). Linear discriminant analysis (LDA) effect size (LEfSe) based on non-parametric factional Kruskal-Wallis test (alpha value of 0.05) was used to detect the features of bacterial communities at multiple taxonomical levels. The threshold of LDA score for discriminative biomarkers was 2.0^59^.

## Supplementary information

supplementary Informations
