# Supplementary material for: Short-term response of the soil bacterial community to differing wildfire severity in Pinus tabulaeformis stands
Source: Sci Rep. 2019 Feb 4;9:1148. doi: 10.1038/s41598-019-38541-7 (PMC6362210; doi:10.1038/s41598-019-38541-7)

**Short-term response of the soil bacterial community to differing wildfire severity in** ***Pinus tabulaeformis* stands**

Weike Li^1^, Shukui Niu^1^, Xiaodong Liu^1^**^*^**, Jianming Wang**^1^**

1. The College of Forestry, Beijing Forestry University, No. 35 Qinghua East Road, Haidian District, Beijing 100083, China.

* Corresponding author

Telephone number: +8601062336703. E-mail address: xd_liu@bjfu.edu.cn

Table S1 Soil physical and chemical properties.

| Indices | Soil depth/cm | Fire severity | | | |
| --- | --- | --- | --- | --- | --- |
|  |  | H | M | L | C |
| OM /(mg·kg^-1^) | 0-10 | 9190±90b | 17130±760a | 17460±1230a | 16160±550a |
|  | 10-20 | 4000±490b | 6910±590a | 7390±1180a | 7920±440a |
| TN/ (mg·kg^-1^) | 0-10 | 310±10c | 560±40b | 850±30a | 880±50a |
|  | 10-20 | 180±10d | 270±40c | 400±10b | 660±40a |
| NH_4_^+^-N/(mg·kg^-1^) | 0-10 | 1.72±0.03c | 1.45±0.37c | 3.70±0.62 b | 19.49±0.90a |
|  | 10-20 | 1.25±0.30c | 3.01±0.68c | 11.78±0.67b | 16.32±1.07a |
| NO_3_^-^-N/ (mg·kg^-1^) | 0-10 | 1.64±0.43b | 2.45±0.84a | 3.56±0.36a | 2.64±0.06a |
|  | 10-20 | 2.10±0.62b | 2.10±0.44b | 3.87±0.72a | 3.16±0.54a |
| SM/ (%) | 0-10 | 17.23±1.20a | 10.22±1.44c | 11.04±1.31c | 13.43±0.68b |
|  | 10-20 | 11.39±0.10b | 7.21±0.68c | 11.25±0.99b | 15.41±0.66a |
| pH | 0-10 | 6.90±0.27a | 6.13±0.12b | 5.68±0.38b | 5.65±0.25b |
|  | 10-20 | 6.19±0.03a | 5.67±0.14a | 5.62±0.49a | 5.61±0.44a |

OM, organic matter; TN, total nitrogen; NH_4_^+^-N, ammonium nitrogen; NO_3_^-^-N, nitrate nitrogen; SM, soil moisture. H, high severity; M, moderate severity; L, low severity; C, unburned. Different lowercase letters after entries indicate significant differences at the same soil depth of different sample plots at *P*<0.05. Values are means ±standard deviations (*n*=3).

Table S2 Relationship (r) between the dominant phyla and soil variables determined by Pearson Correlation analysis.

| 0-10cm | OM | TN | NH_4_^+^-N | NO_3_^-^-N | SM | pH |
| --- | --- | --- | --- | --- | --- | --- |
| Acidobacteria | 0.233 | 0.404 | 0.221 | 0.194 | -0.034 | -0.188 |
| Proteobacteria | -0.477 | -0.689* | -0.368 | -0.424 | 0.239 | 0.508 |
| Actinobacteria | 0.673* | -0.329 | -0.538 | 0.005 | -0.378 | 0.129 |
| Verrucomicrobia | 0.126 | 0.538 | 0.716** | 0.032 | 0.107 | -0.620* |
| Chloroflexi | 0.427 | 0.659* | 0.550 | 0.399 | -0.142 | -0.611* |
| 10-20cm |  |  |  |  |  |  |
| Acidobacteria | -0.508 | -0.288 | -0.419 | -0.291 | 0.102 | 0.584* |
| Proteobacteria | 0.449 | 0.250 | 0.244 | -0.027 | -0.221 | -0.604* |
| Actinobacteria | 0.243 | -0.163 | 0.082 | 0.268 | -0.316 | -0.134 |
| Verrucomicrobia | -0.052 | 0.299 | 0.180 | 0.058 | 0.447 | -0.025 |
| Chloroflexi | -0.108 | -0.236 | -0.152 | 0.027 | -0.071 | 0.285 |

*Difference is significant at *P*<0.05, ** difference is significant at *P*<0.01.

Table S3 Criteria for the classification of forest fire severity.

| Fire severity level | Criteria for the classification |
| --- | --- |

| H | Trees more than 80% burns or burned to death, the undergrowth shrubs all burned down, char height of trunks were more than 5 m. As a result of the ground organic matter all burned, mineral soil color and structure are changed． |
| --- | --- |
| M | Between height and low-grade fire. The upper part of forest litter was burned. |
| L | Trees were burned less than 10%, part of the undergrowth shrubs was burned down (below 40%), char height of trunks were below 2 m. |
| C | unburned |

Figure S1 Location of the study area in Pingquan County, Hebei province, China.





Figure S2 The plots of linear discriminant analysis (LDA) to show differentially bacterial taxa as biomarkers determined using Kruskal-Wallis test (p<0.05) with LDAscore >2.0.


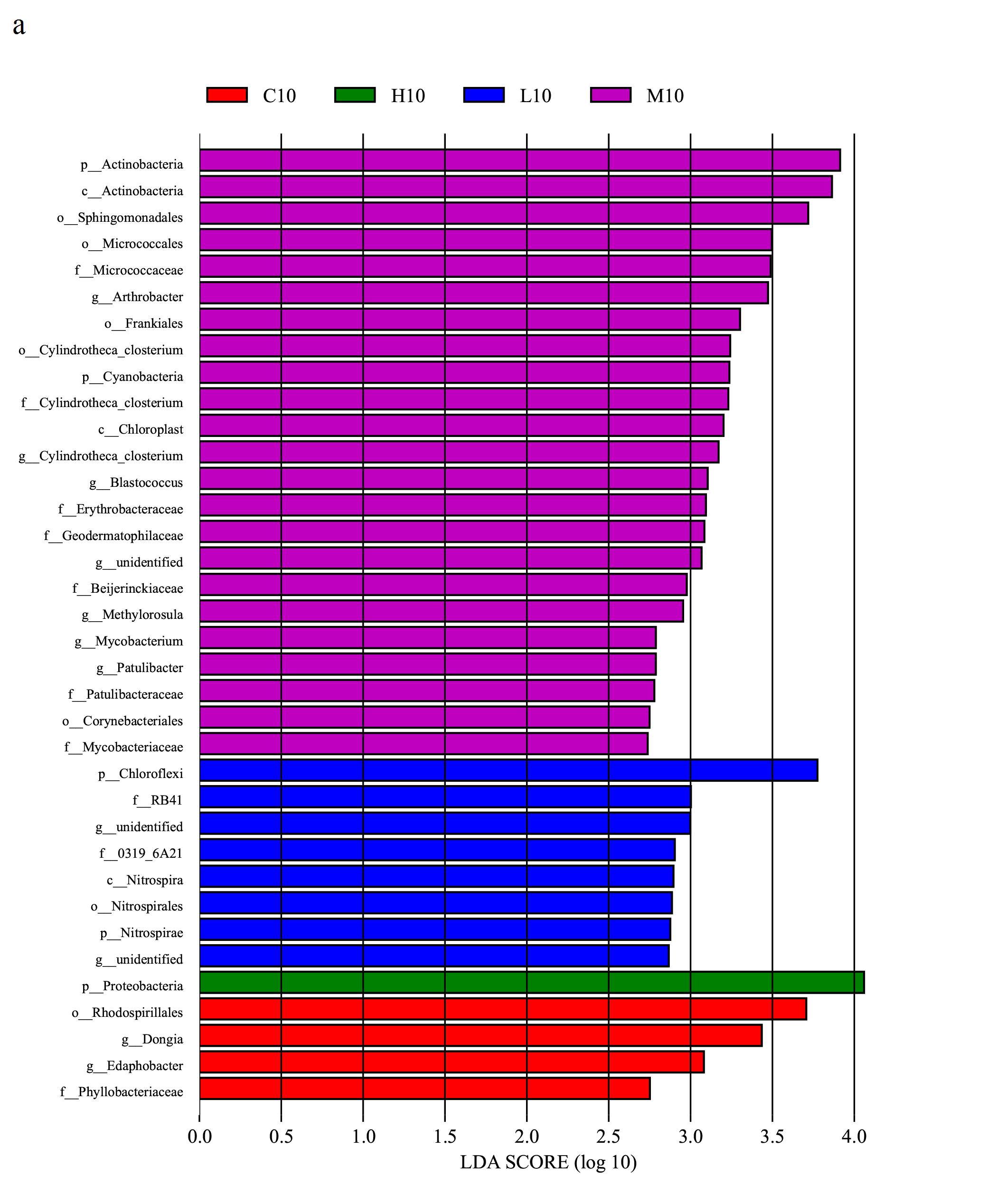


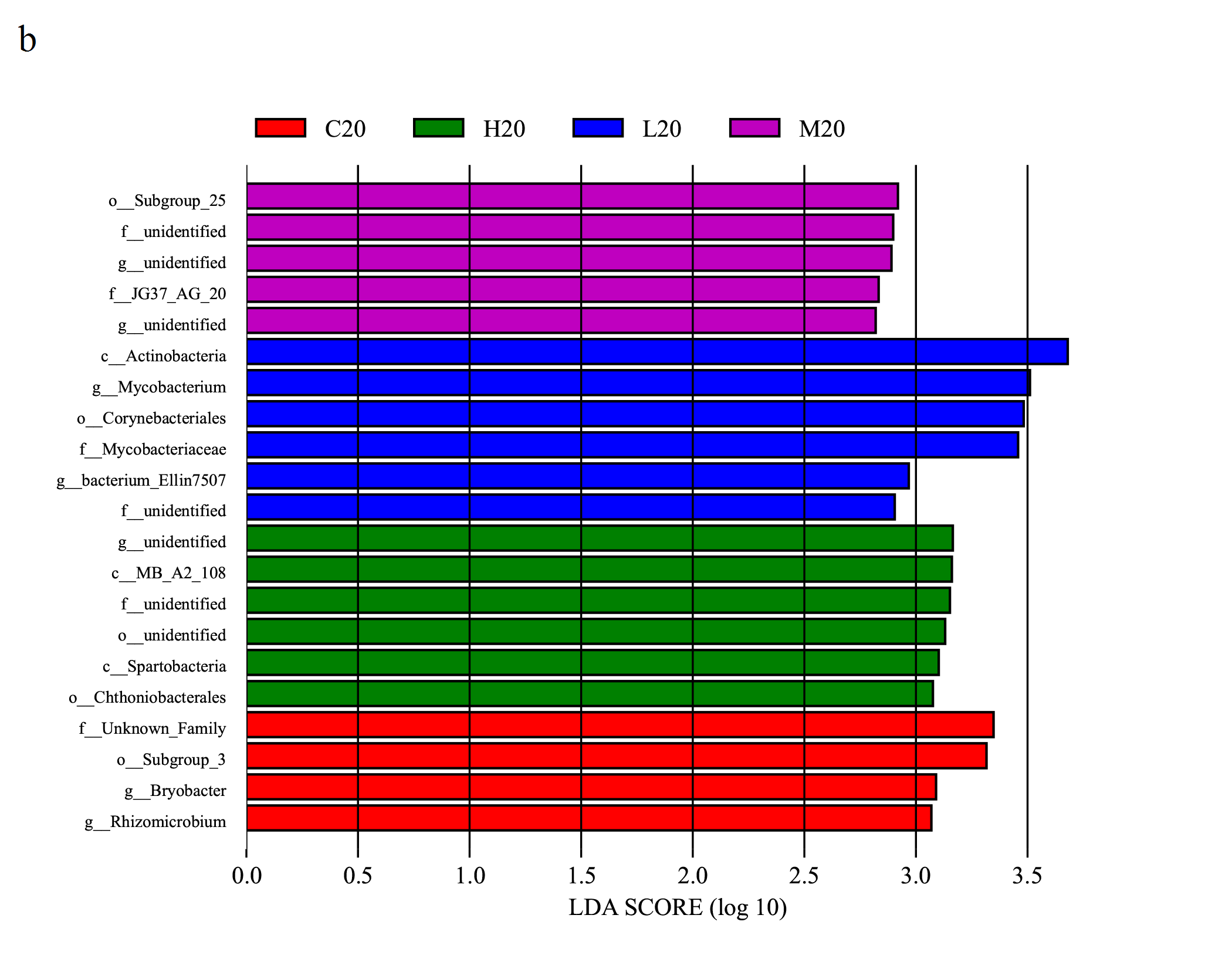

Supplement: Supplementary file 1 — supplementary Informations [file 41598_2019_38541_MOESM1_ESM.docx]
